# Supplementary material for: Production of Recombinant Zika Virus Envelope Protein by Airlift Bioreactor as a New Subunit Vaccine Platform
Source: Int J Mol Sci. 2023 Sep 11;24(18):13955. doi: 10.3390/ijms241813955 (PMC10531330; doi:10.3390/ijms241813955)
Supplement: Supplementary file 1 [file ijms-24-13955-s001.zip › ijms-2265152-supplementary.pdf]

Supplementary File S1: Cytokine primers sequences

Primer gene	Forward Primer	Reverse Primer
IL-1β	CAACCAACAAGTGATATTCTCCATG	GATCCACACTCTCCAGCTGCA
IL-2	CCTGAGCAGGATGGAGAATTACA	TCCAGAACATGCCGCAGAG
IL-4	ACAGGAGAAGGGACGCCAT	GAAGCCCTACAGACGAGCTCA
IL-5	AGCACAGTGGTGAAAGAGACCTT	TCCAATGCATAGCTGGTGATTT
IL-6	GAGGATACCACTCCCAACAGACC	AAGTGCATCATCGTTGTTCATACA
INF-γ	TCAAGTGGCATAGATGTGGAAGAA	TGGCTCTGCAGGATTTTCATG
TGF-β	TGACGTCACTGGAGTTGTACGG	GGTTCATGTCATGGATGGTGC
IL-10	GGTTGCCAAGCCTTATCGGA	ACCTGCTCCACTGCCTTGCT
IL-17	ACCGCAATGAAGACCCTGAT	TCCCTCCGCATTGACACA
IL-35	CCTTTGTGGCTGAGCGAATC	CACCTGGCGGAAGTGAGA
GM-CSF	GCCATCAAAGAAGCCCTGAA	GCGGGTCTGCACACATGTTA
GAPDH	CCTTCCGTGTTCCTACCC	GCCCTCAGATGCCTGCT

Supplementary File 1. Primer sequences for mRNA expression of IL-1β, IL-6, GM-CSF, IL-2, IL-4, IL-17, IFN-γ, IL-10, IL-35, and TGF-β.
